# Exploring the Relation: Does Forgiveness Enhance Interpersonal Problem Solving?

**DOI:** 10.3390/bs15010035

**Published:** 2025-01-01

**Authors:** Çağla Girgin Büyükbayraktar, Süleyman Barbaros Yalçın, İsmail Yavuz Öztürk, Serkan Say

**Affiliations:** 1 Department of Social Work, Beyşehir Ali Akkanat School of Applied Sciences, Selçuk University, Konya 42700, Turkey; 2 Department of Educational Sciences, Guidance and Psychological Counseling Program, Ahmet Kelesoglu Faculty of Education, Necmettin Erbakan University, Konya 42090, Turkey; barbarosyalcin@erbakan.edu.tr; 3 Department of Turkish Education, Faculty of Education, Mersin University, Mersin 33343, Turkey; iyavuzozturk@mersin.edu.tr; 4 Department of Classroom Education, Faculty of Education, Mersin University, Mersin 33343, Turkey; serkansay@mersin.edu.tr

**Keywords:** forgiveness, interpersonal problem solving, university students, stress management, social support

## Abstract

This study explores the relationship between forgiveness and interpersonal problem-solving skills among university students using a correlational design. The sample includes 443 students aged 18–26 from Mersin and Selçuk Universities, selected through convenience sampling. Data were collected using the Heartland Forgiveness Scale, the Interpersonal Problem-Solving Inventory, and a personal information form. Pearson correlation and multiple regression analyses were conducted. Results indicate significant relationships between forgiveness and problem-solving skills. Forgiveness positively predicts constructive problem-solving (r = 0.45, *p* < 0.01) and negatively correlates with negative approaches to problems (r = −0.37, *p* < 0.01), lack of self-confidence (r = −0.29, *p* < 0.01), and unwillingness to take responsibility (r = −0.31, *p* < 0.01). Forgiveness explains 25.2% of the variance in negative approaches, 8% in constructive problem-solving, 13.4% in self-confidence, and 10.3% in responsibility avoidance. Self-forgiveness, forgiveness of others, and situational forgiveness are significant predictors across these dimensions. Findings suggest that forgiving students manage interpersonal conflicts more positively, with reduced negative emotions and avoidance behaviors. Promoting forgiveness and problem-solving skills through educational programs may enhance students’ social harmony, adjustment, and life satisfaction, benefiting both individuals and society. This aligns with existing literature highlighting the emotional and relational benefits of forgiveness.

## 1. Introduction

There are many stress factors for students who continue their university education, such as academic issues, social relationships, and personal development (Alkhawaldeh et al., 2023). Individuals can mitigate the negative effects of these stressors and subsequent perceived stress by using various adaptive strategies such as forgiveness, social support, and resilience when coping with interpersonal and psychological stress (Dohrenwend, 2000). Interpersonal problem-solving skills can be an effective tool in coping with these stressors (Abdollahi et al., 2016).

Problems experienced in interpersonal relationships can negatively affect students’ academic performance (Peel & Ward, 2022). Forgiveness and interpersonal problem-solving skills can have a positive effect on students’ academic success via providing them with the ability to deal with such problems more effectively (Chan, 2022).

In addition to all this, university years are a period when individuals interact with different people and develop various social skills (Khasanzyanova, 2017). Responding forgivingly in conflict situations that they may encounter during this period supports students in establishing healthier and more sustainable relationships in terms of reconciling both parties of the conflict (Aquino et al., 2006), and is a problem-solving and coping strategy that reconciles the conflicting parties and saves the social relationship for future interactions (Aquino et al., 2003).

Forgiveness and problem-solving skills can contribute to the personal development of individuals. Forgiveness promotes characteristics such as empathy (Aprilia & Nashori, 2023) and patience (Ahmadi et al., 2023), while problem-solving skills develop abilities such as analytical thinking (Robbins, 2011), creativity (Orakci, 2023), and effective communication (Maksum et al., 2024).

Forgiveness is considered a widely encouraged problem-solving strategy (Butler & Mullis, 2001). As an adaptive coping mechanism, forgiveness requires softening the evaluation of events (Rahmandani & Amaranggani, 2023). The forgiveness and problem-solving skills developed during college years can also play an important role in students’ future professional and personal lives (Dahiya & Rangnekar, 2019; Antıć & Bogetıć, 2024). These skills can serve as important tools for coping with challenges in the workplace and in personal relationships. Forgiveness and interpersonal problem solving are significant in social interactions, raising questions about whether such problem solving improves when individuals who have forgiven each other interact. Although interpersonal problem solving has been widely studied over the past two decades, research on outcomes influenced by forgiveness remains limited. Recent studies indicate this is a promising area for further exploration. Beyond personal relevance for those with religious or philosophical beliefs, there is a growing body of social science research on forgiveness, especially in clinical, familial, marital, and intercultural contexts (Alnawas et al., 2022; Dehdashti-Lesani et al., 2021; Stephanou & Giorgali, 2020).

Developing forgiveness and interpersonal problem-solving skills in university students could produce positive results both at the individual and societal levels in this context.

The aim of this paper is to examine the relationship between forgiveness and interpersonal problem solving among university students. The following questions were sought:

1. Is there a significant relationship between forgiveness and interpersonal problem solving among university students?
2. Does forgiveness predict negative approach to problem (NAP) among university students?
3. Does forgiveness predict constructive problem solving (CPS) among university students?
4. Does forgiveness predict lack of self-confidence (LSC) among university students?
5. Does forgiveness predict not taking responsibility (NTR) among university students?

## 2. Literature

### 2.1. Forgiveness

Forgiveness is an action freely chosen by the forgiver (Baskin & Enright, 2004). The person who chooses to forgive avoids the experience of anger or can end the persistence of stress experiences and thoughts of revenge (Lawler et al., 2003). It is also a process that provides positive adaptation by letting go of negative feelings, thoughts, and hatred towards the guilty. In this way, compassion, generosity, and goodness are encouraged towards that person (Enright, 1991). Toprak (2013) emphasized that it is important for experts working in the field of mental health to use the concept of “forgiveness” effectively in counseling, and presented a broad perspective on the subject of forgiveness in the psychological counseling process. Learning to forgive is an important step among the steps taken to increase the quality of life; this process can contribute to improving both physical and mental health (Hallowell, 2004). Ironically, when we forgive, we free ourselves from being slaves to our hatred, based on the liberating meaning of forgiveness (Hallowell, 2004).

Hargrave & Sells (1997) defined forgiveness as follows: Forgiveness is the process of setting aside anger and feelings of revenge toward the guilty, restoring one’s relationships, and healing internal emotional wounds. The concept of forgiveness can be defined as a process that reduces the motivation to seek revenge against the guilty and prevents alienation from the guilty. It is also a series of changes that can increase reconciliation and goodwill despite the offending person’s hurtful actions (McCullough et al., 1998). In forgiveness, a person overcomes his/her anger toward the guilty but does not deny his/her moral right to hold resentment (Subkoviak et al., 1995). What is unique about forgiveness is the focus on morality and goodwill toward others in the act of forgiveness (Wade et al., 2014).

Berry et al. (2005) accepted forgiveness as an interpersonal phenomenon. Forgiveness includes components of compassion, empathy, and sympathy towards others (Worthington et al., 1999) and also regulates interpersonal relationships (Yamhure-Thompson & Snyder, 2003). In forgiveness processes between individuals, it is generally expected that the guilty person will be forgiven by the aggrieved person (Bassett et al., 2006).

Interpersonal forgiveness is a set of process changes in social motivation when individuals encounter an upsetting situation; this type of forgiveness occurs when individuals replace destructive reactions with constructive behaviors in order to achieve reconciliation with others (McCullough et al., 2001). Interpersonal forgiveness consists of an increase in benevolence toward the offender combined with a decrease in revenge and avoidance (McCullough & Hoyt, 2002). Interpersonal forgiveness refers to (a) a decreasing motivation to seek revenge on a partner in an abusive relationship, (b) a decreasing motivation to distance oneself from the guilty, and (c) an increasing motivation to reconcile and have goodwill with the guilty despite the guilty’s hurtful actions (McCullough et al., 1997). It has been observed that when individuals express forgiveness for an interpersonal offense, they increase the likelihood of reestablishing harmonious and positive interpersonal relationships with the offender (McCullough et al., 1997; Özteke-Kozan et al., 2017). It is also stated that forgiveness programs alleviate interpersonal feelings such as aggression, anger, and hostility, and are a method that can be used to reduce aggressive behavior (Skalski-Bednarz, 2024).

Various frameworks define forgiveness in scholarly discourse, primarily through emotional and psychological aspects. Emotional forgiveness involves mitigating feelings of resentment toward an offender, promoting tranquility and well-being. This internal process allows victims to release adverse emotions, especially when the offender is absent, reducing grievances directed at them. As a result, the offender ceases to be viewed as a source of hostility, enhancing the victim’s overall well-being. These dynamics illustrate how individuals engage with their past, often struggling with resentment and their perceived fate (Almeida & Cunha, 2023; Brady et al., 2022; Kamomoe et al., 2021).

### 2.2. Interpersonal Problem Solving

Human relationships are the main source of both happiness and unhappiness. Therefore, many problems in life arise from interpersonal relationships. Difficulties brought about by relationships with other people can lead to individual problems (Eskin, 2014). On the other hand, acquiring and applying positive interpersonal skills is very important for developing and maintaining successful interpersonal relationships (Wilson, 1981).

As long as an inconsistency is perceived, there is a problem (Deno, 2005). A problem is any life situation or task that demands an effective response to achieve a goal or resolve a conflict when no effective response is immediately apparent or appropriate to a person (D’Zurilla & Nezu, 2010). A problem can be defined as the difference between the current state of something and its ideal or expected state, or the difference between the current state of things and their intended state (Kneeland, 2001). A problem is something that is placed in front of us or hinders us (Folkman, 2010; Kalaycı, 2001). Problem solving is the process of overcoming the difficulties encountered in reaching a goal (Bingham, 1998) and finding solutions to the difficulties encountered in reaching a goal (Çam & Tümkaya, 2006). Problem solving is the process of overcoming the difficulties encountered in reaching a goal (Bingham, 1998) and finding solutions to the difficulties encountered in reaching a goal (Çam & Tümkaya, 2006). Most problem-solving models usually have five stages: general orientation, problem identification, generation of alternatives, decision making, and evaluation (Heppner & Petersen, 1982).

According to Nezu (2004), social problem solving is the cognitive-behavioral process used to find successful solutions to everyday problems. The successful resolution of interpersonal problems is an important means of behavior modification. When individuals with different goals, temperaments, and experiences interact, the need for effective problem solving will arise (Feldgaier, 1987). Problem solving style refers to the cognitive and behavioral activities by which a person tries to understand problems in life and find effective solutions or ways to cope with them (Bell & D’Zurilla, 2009).

Individuals face various barriers in resolving interpersonal conflicts, such as personal biases, perception patterns, emotional states, lack of information, and goal-setting processes. Examining the connection between forgiveness and problem-solving in interpersonal relationships provides a fresh perspective on their dynamics, which is the central theme of this study. Optimizing solutions for relationship management is vital since interpersonal relationships are essential in life. Effectively managing conflict is crucial for resolution and forgiveness. Therefore, understanding the sequence of problem-solving activities and emotional responses during crises is key. A thorough exploration of causal relationships linking problem-solving with forgiveness outcomes is crucial (Parsakia et al., 2023; Martinez-Díaz et al., 2021; Stephanou & Giorgali, 2020).

## 3. Research Method

Revealing whether there is a relationship between forgiveness and interpersonal problem solving among university students was aimed at in this research. A correlational design, to be used to examine and describe the relationship between variables and to obtain information about the relationship, dependency, or relationship level between variables (Creswell, 2013), was used within the scope of quantitative research.

### 3.1. Study Group

Study group of this research consists of students studying at different faculties of Mersin University and Selçuk University. The convenience sampling method, which is not probability-based sampling method, was used when determining the sample. This selected sampling method is expected to consist of volunteers and individuals who are likely to be reached (Stratton, 2021; Fraenkel & Wallen, 2006). The study group created with this sampling method consists of 443 university students. Descriptive findings of the study group of the research are given in Table 1.

### 3.2. Data Collection Tools

#### 3.2.1. Interpersonal Problem-Solving Inventory (IPSI)

The inventory, developed by Çam and Tümkaya (2006) to measure the interpersonal problem-solving orientation and skills of university students, consists of a total of 50 items. These items are evaluated using a five-point, Likert-type scale, with evaluation options ranging from “Not at all appropriate” (1) to “Completely appropriate” (5). High scores obtained in subscales indicate that individuals have high interpersonal problem-solving characteristics. Exploratory factor analysis was performed on 526 individuals and as a result of this analysis, five factors were identified that explained 38.38% of the variance related to interpersonal problem solving. These factors are named as follows according to their content: NAP, CPS, LSC, NTR, Insistent-Persistent Approach (I-PA). The number of items in each subscale are 16, 16, 7, 5, and 6, respectively (Çam & Tümkaya, 2006).

#### 3.2.2. Heartland Forgiveness Scale (HFS)

The scale, developed by Thompson et al. (2005), aims to measure the levels of self-, other-, and situational forgiveness of university students. This scale consists of 18 items with 7-point Likert-type scores (1: Does not reflect me at all, 7: Completely reflects me) and three sub-dimensions (self-forgiveness, forgiveness of others, and situational forgiveness). The study to adapt the scale to Turkish culture was carried out by Bugay and Demir (2010). Items 1, 2, 3, 4, 5, and 6 in the scale represent the sub-dimension of self-forgiveness (SF); items 7, 8, 9, 10, 11, and 12 represent the sub-dimension of forgiveness towards others (FO); and items 13, 14, 15, 16, 17, and 18 represent the sub-dimension of situational forgiveness (SitF). The scores that can be obtained from the scale vary between 18 and 126. Items 2, 4, 6, 7, 9, 11, 13, 15, and 17 are reverse scored in the scale scoring. The internal consistency reliability of the Turkish form was evaluated with Cronbach’s alpha coefficients: 0.64 for the SF subscale, 0.79 for the FO subscale, and 0.76 for the SF. Cronbach alpha value for the entire scale was calculated as 0.81. Exploratory and confirmatory factor analyses were conducted to test the suitability of the original three-factor structure of the scale for the Turkish sample. According to the results of the Confirmatory Factor Analysis, it was determined that the fit values of the 18-item form of the scale and its three sub-dimensions (SF, FO, SitF) were at a sufficient level according to the research data: GFI = 0.92, AGFI = 0.90, RMSEA = 0.06 (Bugay & Demir, 2010).

#### 3.2.3. Personal Information Form

The personal information form prepared by the researchers was used to determine the gender and age of the participants in the study.

### 3.3. Data Collection

Data collection tools were applied face to face to students who volunteered to respond. Explanations were provided in cases where students did not understand, had difficulty, or requested clarification.

### 3.4. Data Analysis Method

After the data were collected, the scales that were filled in incompletely were excluded from the scoring. The data were analyzed using the SPSS 24 software. Pearson product-moment correlation analyses were conducted to determine the relationships between forgiveness and interpersonal problem solving. Multiple linear regression analyses were conducted to determine the predictive role of forgiveness dimensions on interpersonal problem solving. Before conducting multiple linear regression analysis, the assumptions of the analysis were tested. For this purpose, the relationships between the predictor variables were examined to determine whether there was a multicollinearity problem. For all research questions, the predictor variables were SF, FO, and SitF. The correlation coefficients between these variables ranged from 0.194 to 0.483. Low to medium level correlations between predictor variables indicate that there is no multicollinearity. In addition, VIF and Tolerance values were examined and VIF < 10 and Tolerance > 0.20 were found for all analyses. Durbin-Watson statistics were also used to determine whether there was an autocorrelation problem. Durbin-Watson values were obtained between 1.5 and 2.5 for all analyses. Therefore, there is no autocorrelation problem. Finally, whether the residual values were normally distributed or not was examined with the QQ plot and it was understood that the residual values were also normally distributed. In this direction, multiple linear regression analysis was used.

### 3.5. Limitations

The use of a convenience sampling method limits the generalizability of the results. As the study employed a cross-sectional design, causal relationships cannot be inferred. Additionally, reliance on self-report measures introduces the risk of social desirability bias.

## 4. Results

In this section, the results and comments of the analyses carried out within the scope of the research objectives are included (Table 2).

A Cronbach alpha value above 0.70 indicates that it is quite reliable, and 0.60 and above indicates that it is at an acceptable level of reliability. Skewness and Kurtosis values should be between −2 and +2 for a normal distribution.

The mean, standard deviation, and minimum and maximum values of the HFS and IPSI sub-dimensions are given in Table 3. These data reveal that the participants’ forgiveness and problem-solving skills show a wide distribution.

Table 4 to be contained the correlation coefficients, shows that there are statistically significant relationships between the HFS scores and the scores of NAP, CPS, LSC, and NTR (*p* < 0.05). However, I-PA scores do not show a statistically significant relationship with any of the sub-dimensions of the HFS. (*p* > 0.05). For this reason, the regression model planned to be tested between these variables was not tested.

As seen in Table 5, forgiveness scores explain 25.2% of the variance in NAP (F_3,432_ = 48.159; *p* < 0.05). SF, FO, SitF scores are statistically significant predictors of NAP scores (*p* < 0.05). Accordingly, the following regression equation can be established:

NAP = 76.925 − 0.709 × SF + 0.210 × FO − 0.788 × SitF + *error*

NAP: Negative Approach to Problem; SF: Self-Forgiveness; FO: Forgiveness towards Others; SitF: Situational Forgiveness.

Regression equation to be found reveals that a decrease of 0.709 units is expected with a one-unit increase in the SF variable; a decrease of 0.210 units with a one-unit increase in FO scores and a decrease of 0.788 units with a one-unit increase in SitF scores in NAP scores. In addition, according to the standardized regression coefficients, the order of importance of the predictor variables in explaining the variable of approaching the problem negatively is SitF, SF, and FO, from the most important to the least important, respectively.

Forgiveness scores explain 8% of the variance in the CPS (F_3,432_ = 12.353; *p* < 0.05) as seen in Table 6. Among the variables included in the analysis, only SitF scores are a statistically significant predictor of CPS scores (*p* < 0.05). Accordingly, the following regression equation can be established:

CPS = 39.732 + 0.436 × SitF + *error*

CPS: Constructive Problem Solving; SitF: Situational Forgiveness

Regression equation to be found indicates that a one-unit increase in SitF scores is expected to result in a 0.436 unit increase in CPS.

Table 7 brings out that forgiveness scores explain 13.4% of the variance related to LSC (F_3,432_ = 22.060; *p* < 0.05). SF and SitF scores are statistically significant predictors of LSC scores (*p* < 0.05). Accordingly, the following regression equation can be established:

LSC = 23.043 − 0.176 × SF − 0.225 × SitF

LSC: Lack of Self-Confidence; SF: Self-Forgiveness; SitF: Situational Forgiveness

A decrease of 0.176 units is expected in LSC scores with a one-unit increase in SF scores and a decrease of 0.225 units is expected with a one-unit increase in SitF scores according to this regression equation. As to the standardized regression coefficients, of these two predictor variables, SitF has a more important place in the regression model than SF.

Forgiveness scores explain 10.3% of the variance in NTR (F_3,432_ = 16.355; *p* < 0.05) as seen in Table 8. SF, FO and SitF scores are statistically significant predictors of NTR scores (*p* < 0.05). Accordingly, the following regression formula can be derived:

NTR = 19.702– 0.081 × SF − 0.105 × FO − 0.107 × SitF + *error*

NTR: Not Taking Responsibility; SF: Self-Forgiveness; FO: Forgiveness towards Others; SitF: Situational Forgiveness.

When the regression equation obtained is examined, a decrease of 0.081 units is expected with a one-unit increase in the SF variable in NTR scores; a decrease of 0.105 units with a one-unit increase in FO scores; and a decrease of 0.107 units with a one-unit increase in SitF scores. In addition, according to the standardized regression coefficients, the order of importance of the predictor variables in explaining the NTR variable from the most important to the least important is FO, SitF and SF, respectively.

## 5. Discussion

Upon examining the correlation coefficients as to the research sub-question, “Is there a significant relationship between forgiveness and interpersonal problem-solving among university students?”, statistically significant relationships were identified between HFS scores and NAP, CPS, LSC, and NTR scores (*p* < 0.05). However, I-PA scores did not show a statistically significant relationship with any of the HFS sub-dimensions (*p* > 0.05). Therefore, the regression model planned to be tested between these variables was not tested.

A statistically significant relationship was found between HFS scores and NAP. This may indicate that individuals with low forgiveness approach problems more negatively. Forgiveness plays an important role in individuals’ emotional regulation (Ho et al., 2020; Chilwal & Gautam, 2024), and it can be assumed that more forgiving individuals approach problems more positively and constructively (McCullough et al., 1997; Karremans et al., 2003). The result of a significant relationship between CPS and forgiveness may indicate that forgiving individuals adopt more constructive strategies to solve problems. Forgiveness can be an attitude that produces positive outcomes in interpersonal relationships (Flanagan et al., 2012), helping individuals deal with problems more effectively (McCullough et al., 2011). The result of a significant relationship between LSC and forgiveness may suggest that forgiveness is related to self-confidence. Forgiving individuals may have more self-confidence (Turnage et al., 2012), and this confidence may be positively reflected in problem-solving processes. The result of a significant relationship between forgiveness and NTR may indicate that forgiving individuals are more willing to take responsibility. Forgiveness can help individuals accept their own mistakes and the mistakes of others and take responsibility for these situations (Fisher & Exline, 2006). The fact that there was no significant relationship between I-PA scores and any of the sub-dimensions of the HFS may indicate that these two variables are independent of each other. This may suggest that I-PA is not affected by forgiveness or that forgiveness has no effect on this approach. These results suggest that forgiveness may promote positive problem-solving strategies and reduce negative approaches, but forgiveness does not appear to have an effect on certain strategies, such as I-PA. These results are important for understanding the role of forgiveness in individuals’ interpersonal relationships (Tsang & Martin, 2018) and how this role is reflected in problem-solving processes (Massey, 2009). While studies supporting the research are (Thompson et al., 2005; Worthington et al., 2007), studies opposing the research are Luchies et al. (2010; Kachadourian et al., 2005).

Forgiveness scores in relation to the sub-question of “Does forgiveness predict negative approach to problem among university students?” explain 25.2% of the variance in approaching the problem negatively (F_3,432_ = 48.159; *p* < 0.05). SF, FO, SitF scores are statistically significant predictors of NAP scores (*p* < 0.05). A one-unit increase in SF variable is expected to result in a 0.709-unit decrease; a one-unit increase in FO scores is expected to result in a 0.210-unit increase; and a one-unit increase in SitF scores is expected to result in a 0.788-unit decrease in NAP scores. In addition, according to standardized regression coefficients, the order of importance of predictor variables in explaining the NAP variable is SitF, SF, and FO, from most to least important.

This paper examined the extent to which university students’ forgiveness levels predicted their NAP. The results showed that forgiveness scores explained 25.2% of the variance related to NAP. This suggests that forgiveness significantly affects NAP and is a significant predictor (Thomas & Kamble, 2023). Specifically, SF, FO, and SitF scores were each a significant predictor of NAP scores. These results suggest that forgiveness plays an important role in interpersonal problem-solving processes and is effective in reducing NAP (Karremans & Van Lange, 2004). These results reveal how different dimensions of forgiveness (SF, FO, and SitF) play different roles in interpersonal problem-solving processes. SF and SitF appear to be particularly effective in reducing NAP, while FO appears to have different dynamics and is less effective in reducing NAP. These results suggest that interpersonal relationships and problem-solving skills can be improved through the development of forgiveness (Li et al., 2021).

Forgiveness scores for the sub-question “Does forgiveness predict constructive problem solving among university students?” explain 8% of the variance in the CPS (F_3,432_ = 12.353; *p* < 0.05). Of the variables included in the analysis, only SitF scores were a statistically significant predictor of CPS scores (*p* < 0.05). A one-unit increase in SitF scores would be expected to result in a 0.436-unit increase in CPS.

Forgiveness is the ability of individuals to forgive negative situations or people they encounter (Worthington et al., 2007; Long et al., 2020). CPS is the ability of individuals to effectively and positively solve the problems they encounter (Noreen & Dritschel, 2023). In this study, forgiveness was found to predict CPS. This suggests that forgiving individuals tend to resolve problems more constructively (Lathren et al., 2021; McNulty & Dugas, 2019). Forgiveness can help individuals lighten their emotional burden and adopt a more rational and solution-oriented approach (Worthington et al., 2007; Tsang et al., 2006). Forgiveness scores explained 8% of the variance in CPS, indicating that the role of forgiveness on CPS is limited but significant. This result indicates that other factors also play an important role in CPS, but forgiveness is also an important factor to consider (Fincham & Beach, 2007; Worthington & Scherer, 2004). These results suggest that forgiveness has a positive effect on CPS skills in university students. This suggests that developing students’ forgiveness skills may help them solve problems they encounter more effectively and constructively (Türkoğlu, 2014). Forgiveness can reduce the emotional burden on individuals and enable them to think more objectively and rationally. Studies supporting the results are those of Toussaint et al. (2001; Karremans et al., 2003), while studies opposing these results are those of Fincham et al. (2006) and McNulty (2011).

Forgiveness scores for the sub-question of “Does forgiveness predict lack of self-confidence among university students?” explain 13.4% of the variance in LSC (F_3,432_ = 22.060; *p* < 0.05). SF and SitF scores are statistically significant predictors of LSC scores (*p* < 0.05). A one-unit increase in SF scores would result in a 0.176-unit decrease in LSC scores, and a one-unit increase in SitF scores would result in a 0.225-unit decrease. According to the standardized regression coefficients, SitF has a more important place in the regression model than SF among these two predictor variables.

Forgiveness has been found to predict LSC, indicating that forgiveness has a significant impact on individuals’ self-esteem levels (Maltby et al., 2001; Yamhure-Thompson & Snyder, 2003). Forgiveness can help individuals reduce their negative feelings and self-criticism, which can increase self-confidence (Worthington & Scherer, 2004; Neff & Germer, 2013). Especially for university students, developing forgiveness skills may contribute to the elimination of LSC. The fact that forgiveness scores explain 13.4% of the variance related to LSC shows that the effect of forgiveness on LSC is significant. This result shows that forgiveness is an important factor on LSC (Hall & Fincham, 2005), but there are also other factors that affect LSC. These results suggest that forgiveness, particularly SitF and SF, plays an important role in reducing self-confidence deficits in college students. Educational institutions and psychological counseling services can help students increase their self-confidence by designing programs and interventions to improve their forgiveness skills. Such interventions can contribute to students having more successful and satisfying experiences in both their academic and personal lives.

Forgiveness scores explain 10.3% of the variance in NTR regarding the sub-question of “Does forgiveness predict not taking responsibility among university students?” (F_3,432_ = 16.355; *p* < 0.05). SF, FO and SitF scores are statistically significant predictors of NTR scores (*p* < 0.05). A one-unit increase in the SF variable would result in a 0.081-unit decrease; a one-unit increase in FO scores would result in a 0.105-unit decrease; and a one-unit increase in SitF scores would result in a 0.107-unit decrease in NTR scores. In addition, according to the standardized regression coefficients, the order of importance of the predictor variables in explaining the NTR variable is FO, SitF, and SF, from the most important to the least important, respectively.

Forgiveness has been found to predict NTR. This shows that forgiveness has an impact on individuals’ tendency to take responsibility. Forgiveness enables individuals to forgive themselves and others in the face of negative situations and mistakes (Hall & Fincham, 2005; McCullough et al., 2000), which may increase their tendency to take responsibility. Forgiving individuals can more easily embrace taking responsibility by admitting their mistakes and learning from them. The fact that forgiveness scores explained 10.3% of the variance regarding NTR shows that the effect of forgiveness on NTR is significant. This result indicates that forgiveness is an important factor on NTR (Baumeister et al., 1998), but there are also other factors that affect NTR. Of the variables included in the analysis, SF, FO and SitF scores were found to be statistically significant predictors of NTR scores. These results suggest that forgiveness, especially FO and SitF, plays an important role in enhancing the tendency to take responsibility in university students. Forgiveness is generally considered as a process that increases individuals’ responsibility-taking behaviors and produces more positive outcomes in relationships (McCullough, 2000). People who take responsibility are generally more likely to forgive others (McCullough et al., 2009; Walker & Gorsuch, 2002).

This study makes significant contributions to the existing literature on forgiveness and reveals a noteworthy interpersonal dynamic. However, the idea that forgiveness enhances problem-solving skills does not clearly specify the circumstances or ways in which these skills are utilized, necessitating careful interpretation of the results. Furthermore, recent research indicates that agreeableness plays a crucial role in the relationship between forgiveness and conflict resolution effectiveness, aligning with the results of this study (Dehdashti Lesani et al., 2021; Kaya & Odacı, 2024; Safari et al., 2023).

## 6. Conclusions

In this study, the effect of forgiveness levels of university students on interpersonal problem-solving processes was examined comprehensively. The results revealed statistically significant relationships between HFS scores and NAP, CPS, LSC, and NTR scores. On the other hand, it was determined that I-PA scores did not show a significant relationship with any of the sub-dimensions of the HFS.

Forgiveness scores were found to explain 25.2% of the NAP, 8% of the CPS, 13.4% of the LSC, and 10.3% of the NTR. Analysis results show that SF, FO, and SitF scores are significant predictors of NAP, LSC, and NTR scores. In particular, SitF stands out as the strongest predictor of these three variables, whereas in the CPS, only SitF scores were found to be a significant predictor.

These results indicate that forgiveness significantly affects university students’ problem-solving approaches and self-confidence. Forgiveness plays an important role in reducing NAPs and promoting constructive strategies. However, forgiveness does not appear to have an effect on certain strategies such as I-PA. These results suggest that improving forgiveness can improve students’ interpersonal relationships and problem-solving skills, thereby enabling them to achieve more successful and satisfying life experiences.

These results indicate that forgiveness significantly influences university students’ interpersonal problem-solving approaches and self-confidence. Reduction of Negative Problem-Solving Approaches: Forgiveness, particularly situational forgiveness, appears to play a critical role in helping students manage challenges in a more adaptive and less negative manner. Fostering Constructive Strategies: While forgiveness has a modest impact on constructive problem-solving, its influence through situational forgiveness suggests the importance of context-sensitive forgiveness in interpersonal interactions. Boosting Self-Confidence and Responsibility: Forgiveness contributes to enhanced self-confidence and a greater willingness to take responsibility, potentially aiding students in addressing interpersonal conflicts with greater resilience and accountability. Unexplored Dynamics: Despite these significant relationships, the lack of an association between I-PA scores and forgiveness dimensions raises questions about the contextual or dispositional factors that might mediate or moderate these effects. This suggests that forgiveness may not universally affect all problem-solving strategies and that the relationship may depend on specific interpersonal or situational contexts. Broader Contributions: The results underscore the potential of forgiveness as a psychological resource for enhancing students’ interpersonal relationships and problem-solving skills. Programs that integrate forgiveness-focused interventions could help students develop better conflict resolution strategies, improve self-perception, and strengthen their interpersonal dynamics. Ultimately, these improvements could contribute to more successful and satisfying life experiences. Future Directions: Given the limited impact on CPS and the lack of relationship with I-PA, further research is needed to explore: The role of individual differences, such as personality traits (e.g., agreeableness or openness), in moderating the effects of forgiveness. The influence of contextual factors, such as cultural norms or social support, on how forgiveness translates into problem-solving behaviors. Longitudinal studies to understand the causal pathways between forgiveness, self-confidence, and problem-solving effectiveness over time. In conclusion, this study demonstrates the significant role of forgiveness in shaping university students’ approaches to interpersonal problem-solving. By enhancing forgiveness, students may not only overcome interpersonal challenges more effectively but also foster healthier relationships and a stronger sense of personal agency in their daily lives.

## Figures and Tables

**Table 1:** Data from the study group.

Gender	Frequency (f)	Percentage (%)
Female	240	55.4
Male	193	44.6
Age		
18–20	89	20.6
21–23	294	67.9
24–26	39	9
Ages 27 and Above	11	2.6
Total	443	100

**Table 2:** Internal consistency and normality data for HFS and IPSI scores.

Scale and Sub-Dimensions	Cronbach Alfa (α)	Skewness	Kurtosis
**HFS**			
SF	0.517	0.143	−0.145
FO	0.658	0.005	0.725
SitF	0.626	0.232	0.406
**IPSI**			
NAP	0.906	0.432	−0.377
CPS	0.876	−0.035	−0.076
LSC	0.791	0.907	0.436
NTR	0.688	0.303	−0.463
I-PA	0.738	−0.214	−0.140

HFS: Heartland Forgiveness Scale; SF: Self-Forgiveness; FO: Forgiveness towards Others; SitF: Situational Forgiveness; IPSI: Interpersonal Problem Solving Inventory; NAP: Negative Approach to Problem; CPS: Constructive Problem Solving; LSC: Lack of Self-Confidence; NTR: Not Taking Responsibility; I-PA: Insistent-Persistent Approach.

**Table 3:** Mean scores on HFS and IPSI.

Scale and Sub-Dimensions	Mean	Standard Deviation	Minimum	Maximum
**HFS**				
SF	27.087	5.272	13.00	42.00
FO	25.048	6.201	6.00	42.00
SitF	27.002	5.483	9.00	42.00
**IPSI**				
NAP	41.699	12.730	16.00	78.00
CPS	54.734	9.651	23.00	79.00
LSC	13.466	4.788	7.00	30.00
NTR	12.004	3.992	5.00	24.00
I-PA	20.995	4.322	6.00	30.00

HFS: Heartland Forgiveness Scale; SF: Self-Forgiveness; FO: Forgiveness towards Others; SitF: Situational Forgiveness; IPSI: Interpersonal Problem Solving Inventory; NAP: Negative Approach to Problem; CPS: Constructive Problem Solving; LSC: Lack of Self-Confidence; NTR: Not Taking Responsibility; I-PA: Insistent-Persistent Approach.

**Table 4:** Relationship between HFS and IPSI scores.

	SF	FO	StiF
**IPSI**			
NAP	−0.419 **	−0.119 *	−0.415 **
CPS	0.164 **	0.142 **	0.277 **
LSC	−0.295 **	−0.115 *	−0.318 **
NTR	−0.201 **	−0.255 **	−0.271 **
I-PA	0.055	0.014	0.054

* *p* ˂ 0.05, ** *p* ˂ 0.01. SF: Self-Forgiveness; FO: Forgiveness towards Others; StiF: Situational Forgiveness; IPSI: Interpersonal Problem Solving Inventory; NAP: Negative Approach to Problem; CPS: Constructive Problem Solving; LSC: Lack of Self-Confidence; NTR: Not Taking Responsibility; I-PA: Insistent-Persistent Approach.

**Table 5:** Results of multiple linear regression analysis on forgiveness scores predicting NAP Scores.

NAP									
	*R*	*R* ^2^	*R* ^2^ *ch*	*F*	*Df*	*B*	*β*	*t*	*p*
Intercept	0.502 ^a^	0.252	0.247	48.159	3/432	76.925		22.794	0.000 ^a^
SF						−0.709	−0.294	−6.359	0.000
FO						0.210	0.102	2.148	0.032
SitF						−0.788	−0.340	−6.561	0.000

^a^ Intercept SF, FO, StiF. NAP: Negative Approach to Problem; SF: Self-Forgiveness; FO: Forgiveness towards Others; SitF: Situational Forgiveness.

**Table 6:** Results of multiple linear regression analysis on forgiveness scores predicting CPS Scores.

CPS									
	*R*	*R* ^2^	*R* ^2^ *ch*	*F*	*Df*	*B*	*β*	*t*	*p*
Intercept	0.282 ^a^	0.080	0.073	12.353	3/432	39.732		14.000	0.000 ^a^
SF						0.102	0.056	1.092	0.275
FO						0.018	0.012	0.221	0.825
SitF						0.436	0.248	4.315	0.000

^a^ Intercept SF, FO, SitF. CPS: Constructive Problem Solving; SF: Self-Forgivenes; FO: Forgiveness towards Others; SitF: Situational Forgiveness.

**Table 7:** Results of multiple linear regression analysis on forgiveness scores predicting LSC scores.

LSC									
	*R*	*R* ^2^	*R* ^2^ *ch*	*F*	*Df*	*B*	*β*	*t*	*p*
Intercept	0.366 ^a^	0.134	0.128	22.060	3/432	23.403		17.134	0.000 ^a^
SF						−0.176	−0.193	−3.892	0.000
FO						0.036	0.047	0.912	0.362
SitF						−0.225	−0.258	−4.633	0.000

^a^ SF, FO, SitF. LSC: Lack of Self-Confidence; SF: Self-Forgiveness; FO: Forgiveness towards Others; SitF: Situational Forgiveness.

**Table 8:** Results of Multiple Linear Regression Analysis Regarding the Prediction of Forgiveness Scores on NTR Scores.

NTR									
	*R*	*R* ^2^	*R* ^2^ *ch*	*F*	*Df*	*B*	*β*	*t*	*p*
Intercept	0.321 ^a^	0.103	0.097	16.355	3/432	19.702		16.992	0.000 ^a^
SF						−0.081	−0.106	−2.103	0.036
FO						−0.105	−0.163	−3.124	0.002
SitF						−0.107	−0.147	−2.584	0.010

^a^ SF, FO, SitF. NTR: Not Taking Responsibility; SF: Self-Forgiveness; FO: Forgiveness towards Others; SitF: Situational Forgiveness.

## Data Availability

The data presented in this study are available on request from the corresponding author. The data are not publicly available due to ethical reasons.

## Abbreviations

HFS	Heartland Forgiveness Scale
SF	Self-Forgiveness
FO	Forgiveness towards Others
SitF	Situational Forgiveness
IPSI	Interpersonal Problem Solving Inventory
NAP	Negative Approach to Problem
CPS	Constructive Problem Solving
LSC	Lack of Self-Confidence
NTR	Not Taking Responsibility
I-PA	Insistent-Persistent Approach
